# Secreted trophic factors of mesenchymal stem cells support neurovascular and musculoskeletal therapies

**DOI:** 10.1186/s13287-016-0394-0

**Published:** 2016-09-09

**Authors:** Heidi R. Hofer, Rocky S. Tuan

**Affiliations:** Center for Cellular and Molecular Engineering, Department of Orthopaedic Surgery, University of Pittsburgh School of Medicine, 450 Technology Drive, Room 221, Pittsburgh, PA 15219 USA

**Keywords:** Arthritis, Mesenchymal stem cells, Extracellular vesicles, Endothelial cells, Endothelial cell–mesenchymal stem cell interactions, Neurotrophic activity, Muscle-derived stem cells

## Abstract

Adult mesenchymal stem cells (MSCs) represent a subject of intense experimental and biomedical interest. Recently, trophic activities of MSCs have become the topic of a number of revealing studies that span both basic and clinical fields. In this review, we focus on recent investigations that have elucidated trophic mechanisms and shed light on MSC clinical efficacy relevant to musculoskeletal applications. Innate differences due to MSC sourcing may play a role in the clinical utility of isolated MSCs. Pain management, osteochondral, nerve, or blood vessel support by MSCs derived from both autologous and allogeneic sources have been examined. Recent mechanistic insights into the trophic activities of these cells point to ultimate regulation by nitric oxide, nuclear factor-kB, and indoleamine, among other signaling pathways. Classic growth factors and cytokines—such as VEGF, CNTF, GDNF, TGF-β, interleukins (IL-1β, IL-6, and IL-8), and C-C ligands (CCL-2, CCL-5, and CCL-23)—serve as paracrine control molecules secreted or packaged into extracellular vesicles, or exosomes, by MSCs. Recent studies have also implicated signaling by microRNAs contained in MSC-derived exosomes. The response of target cells is further regulated by their microenvironment, involving the extracellular matrix, which may be modified by MSC-produced matrix metalloproteinases (MMPs) and tissue inhibitor of MMPs. Trophic activities of MSCs, either resident or introduced exogenously, are thus intricately controlled, and may be further fine-tuned via implant material modifications. MSCs are actively being investigated for the repair and regeneration of both osteochondral and other musculoskeletal tissues, such as tendon/ligament and meniscus. Future rational and effective MSC-based musculoskeletal therapies will benefit from better mechanistic understanding of MSC trophic activities, for example using analytical “-omics” profiling approaches.

## Background

From a research, medical, and business standpoint, mesenchymal stem cell (MSC)-based therapies are fascinating. Sales for stem cell products (e.g., as a subset of osteobiologics) were projected to top $600,000,000 by 2015 [1, 2], and a recent Scopus search for musculoskeletal and stem cells resulted in over 3000 documents, with more than a third being reviews. We have limited this review to highlighting noteworthy findings and concepts concerned with the understanding of and challenges with MSC musculoskeletal therapies.

MSCs were discovered in the 1960s [3], named in the early 1990s [4], and purportedly defined by the mid-2000s [5]. Despite the proposed criteria, the functional definition within the literature varies widely. MSCs can be defined by their ability to adhere to tissue culture plastic, their expression of several cell surface molecular epitopes—cluster of differentiation CD73, CD90, and CD105, and others—as well as their lack of several surface markers, including CD45 [6]. Some previously excluded markers are debated within certain circles, such as CD34 and CD146 [7–9]. MSCs can be isolated from a range of tissues, but the most commonly cited sources are bone marrow (BM), adipose tissue, muscle, bone, and perinatal tissues (e.g., Wharton’s Jelly, umbilical vein/cord blood (UV/UCB), and amnion).

While they were originally utilized clinically in hopes of harnessing their differentiation and proliferation potential, MSCs are increasingly thought to also influence, in addition to participating in, tissue function [10, 11], especially within osteochondral spaces [12]. These MSC influences can range from relatively rare activities that require cell contact, such as mitochondrial transfer and cell fusion, to relatively common paracrine MSC actions through extracellular microvesicles or secreted factors. MSCs may modulate the immune response, angiogenesis, apoptosis, oxidation level, migration, and/or differentiation/stimulation of surrounding cells [13]. Because of this alternative use of MSCs, Caplan and Sorell [14] suggested a renaming of MSCs to medicinal signaling cells to suggest a new era of MSC clinical relevance due to their immunomodulatory properties. While acknowledging progress in the other areas mentioned, this work will focus on the current debates concerning sourcing, MSC alterations of angiogenesis, cell differentiation/stimulation, and strategies to improve MSC differentiation.

## Sourcing

Sourcing of MSCs has become an area of debate due to well-recognized potential differences in differentiation abilities and trophic activities of the derived MSCs. Alternatively sourced MSCs may have different differentiation potentials as BM-MSCs, and they may require additional supplementation to achieve robust or similar differentiation. However, while relative abundance and ease of isolation of MSCs may allow their use for successful musculoskeletal interventions [15], there are concerns that diminished numbers of MSCs may be present in BM as patients age or succumb to disease [16]. One study found that mouse MSCs from four common sources (BM, adipose tissue, skeletal muscle, and myocardium) equally supported endothelial cell (EC) network formation in vitro and blood vessel formation in vivo [17]. Muscle-derived stem cells (MDSCs) and satellite cells are thought to contribute to repair of skeletal muscle and bone [18–20]. Although the exact mechanisms remain to be elucidated, cartilage and muscle health has been provocatively linked with changes in or lack of multipotent cell activity, including diseases such as osteoarthritis (OA, cartilage), sarcopenia (muscle), and related muscle and motor neuron diseases [21].

In one study attempting to address the most useful source of MSCs for angiogenesis through a hindlimb ischemia model, Bortolotti et al. examined adipose-derived MSCs (AD-MSCs) and BM-MSCs (along with a subpopulation of CD11-depleted BM-MSCs). They found, as have many others, that MSCs were not incorporated into the healing wound but that wounds, particularly those in muscles, healed more rapidly when exposed to BM-MSCs (regardless of MSC sorting) [22]. Classic proangiogenic, chemotactic, and remodeling molecules were identified as being expressed by MSCs, with several factors appearing prominently in the also effective conditioned medium (CM) (platelet-derived growth factor-B (PDGFB), transforming growth factor beta (TGF-β), stromal-derived factor-1 (SDF1), angiopoietin-1 (Ang1), regulator of G-protein signaling-5 (Rgs5), matrix metalloproteinase-9 (MMP-9), chemokine (C-X-C) ligand-10 (CXCL10), chemokine (C-C) ligand (CCL5)) [22].

Work with 5–6-week-old human embryonic BM-MSCs and MDSCs suggests that MSCs have innate propensities for adipogenic and myogenic differentiation, respectively, which ultimately affects the organization of the engineered tissues [16, 23]. MSCs sourced from older tissue might overcome these propensities depending on the implantation culture environment [24]. Taking a cue from the successful use of stem cells from birth-associated tissues [25], one group recently investigated the angiogenic activity of endometrium/menstrual blood-sourced multipotent cells, showing that they support the recruitment of ECs, blood vessels, and, potentially, the proliferation of hematopoietic stem cells [26].

An encouraging finding is that the number of MSCs required to exert trophic action may be far less than originally calculated as necessary for tissue replacement, because a prospective study of BM-MSC vs mixed BM-MSC + lipoaspirate therapy for OA found no difference in patient-reported outcomes between the two groups [27]. Interestingly, increasing body mass index (BMI) appeared to correlate with patient-reported improvement of function, a link that should be explored in the future [28].

## MSC musculoskeletal clinical use

Evidence for an altered view of MSC efficacy follows results from clinical trials, several of which have recently begun to yield data about long-term MSC efficacy in disease treatment. A large area of MSC-based musculoskeletal research has been directed towards the degenerative joint disease OA, which currently affects approximately 20 million Americans and is projected to affect 20 % of American adults by 2030 [29]. OA is characterized by the degeneration of articular cartilage and synovial inflammation, which alters associated soft tissue and subchondral bone, resulting in bony lesion and osteophyte formation. These degenerative events cause pain and loss of joint mobility and function. Because cartilage has a lower regenerative capacity than other, more vascularized tissues in the body, arthritis and joint degeneration are growing targets of MSC-based therapies.

Recent investigations into MSC treatment of OA have begun to include formal, controlled clinical trials [30] in addition to many uncontrolled trials by private entities. Several international companies, including Cartistem, Regenexx, Regeneus, BioHeart, and Mesoblast, are carrying out phase I and II clinical trials for the treatment of degenerative joint diseases with allogeneic or autologous, multipotent cell types that are capable of mesenchymal differentiation, usually derived from BM or adipose origin [15, 31, 32]. Several other companies have chosen to facilitate MSC isolation within the clinic by constructing machines that quickly sort stem cells from the mixed populations present in surgically isolated tissue [33]. Although published data have been relatively scarce for completed trials, adverse events such as tumors, infections, or premature trial closures have rarely been reported, suggesting safety of MSC-based therapies [34, 35]. The majority of reviewed studies utilize either dissociated cells injected into the joint space or cells delivered via seeding in various biocompatible and/or biodegradable materials.

A recent review details results of nine OA articular cartilage (knee) clinical trials which utilized cultured BM-MSCs, noncultured BM concentrate, peripheral blood-derived stem cells, or cells from the adipose stromal vascular fraction [36]. Some cells were immobilized with hyaluronan, collagen, platelet gel, and/or fibrin glue. Others were injected into the joint or at a defect using only saline. Regardless of cell origin, intra-articular injection of cells (vs hydrogel or flap immobilization through open surgery) resulted in improved clinical function over untreated controls in some studies as long as 5 years post treatment [36]. Pain relief with minor return of function was noted in most studies.

Noting the ameliorative effect of MSCs on joint pain, chronic lower back pain has recently become a target of MSC therapy. Autologous, scaffold-less BM-MSC injection into patients with spinal cord injury in a Brazilian clinical trial suggested clinically meaningful pain relief and possible improvement in cartilage structure after 6 months and as long as 2 years post treatment, although the high number of MSCs utilized coupled with the high cost of the procedure were identified as potential areas for improvement [37]. In a Spanish study, 7 of 12 patients showed mild return of function after 6 months when enrolled in a phase I safety and efficacy study for BM-MSC injection for long-term (>6 months) spinal cord injury [38]; although positive in terms of apparent MSC effect, the study faced almost immediate criticism from other researchers due to the small sample size and lack of appropriate controls [39]. A study by Mesoblast reported decreased lower back pain in 48 % of allogeneic BM-MSC-treated patients vs 13 % in placebo controls up to 2 years post injection [40].

A portion of these analgesic effects could be due to the anti-inflammatory activity of MSCs. Evidence that a decrease in granulocyte macrophage colony-stimulating factor (GM-CSF) resulted from MSC treatment and may decrease disease severity after 4 months comes from a rheumatoid arthritis (RA) clinical trial that used MOR103 antibodies to deplete serum GM-CSF [41]. In an excellent and very recent review of MSC applications to RA, De Bari described how immunomodulation could play a role in RA-specific joint degeneration. Immunoregulators, including interferon gamma (IFN-γ) and tumor necrosis factor alpha (TNF-α), which are regulated through indoleamine 2,3-dioxygenase (IDO) or nitric oxide (NO), and MSC effects on forkhead box p3^+^ (Foxp3^+^) Tregs or CD4^+^ Th17 cells have been suggested [42]. Interestingly, the “transformation hypothesis” proposes that MSCs may become transformed by interplay with chronic inflammatory processes in the joint, resulting in a more aggressive cell type with abilities to either invade the articular cartilage and/or circulate, spreading arthritis to unaffected joints [43, 44]. UV-MSCs may help to relieve the severity of RA symptoms when combined with disease-modifying antirheumatic treatments [45].

Recent exploration of immunomodulation showed that AD-MSC surface-bound glycoprotein A repetitions predomain/leucine-rich repeat containing-32 (GARP/LRRC32), found on CD4^+^/Foxp3^+^ Tregs, megakaryocytes, and platelets, binds to membrane-bound TGF-β1, holding it in an inactivated but readily-accessible state. GARP silencing results in increased secretion and activation of TGF-β1 and impaired proliferation of AD-MSC as well as activation of T cells [46]. Immunosuppressive effects of membrane-bound TGF-β1, especially when bound to extracellular vesicles (EVs), have also been reported for other MSC types [47–49].

## Mechanisms of MSC trophic activity

Insight into the mechanisms of MSC trophic activity is advancing across multiple fields (Table 1). Within the joint space, the MSC secretome is thought to influence the anabolic tendencies of chondrocytes, chondrocyte progenitor cells (CPCs), cartilage-derived stem/progenitor cells (CSPCs), synovium-resident multipotent progenitor cells, osteoblasts/osteoclasts/resident MSCs within the subchondral bone (especially after microfracture), and chondrogenic cells within the infrapatellar fat pad [36, 50, 51]. The MSC secretome can be modified through permanent or temporary alterations. Several studies have found that the exposure of MSCs to proinflammatory factors, sometimes for as little as a few hours, can alter the gene and protein expression of MSCs for days afterwards [52]. Factors known to be secreted or bound to MSC membranes with anti-inflammatory activities (activation of Tregs/tolerogenic dendritic cell phenotype; pro-resolving/M2 macrophage activation; inhibition or proapoptosis of T cells, B cells, NK cells, or dendritic cells; decreasing cytokine production) include: purines, bone morphogenetic proteins (BMPs, specifically BMP-4), CD274, CCL2, Connexin 43, cyclooxygenase (COX)/prostaglandin (PG), CD95/CD95 ligand, galectins, heme oxygenase-1, human leukocyte antigen-G (HLA-G), IDO/kynurenine, interleukin-6 (IL-6), leukemia inhibitory factor (LIF), NO, TGF-β, tumor necrosis factor-inducible gene-6 (TSG6), and vascular endothelial growth factor (VEGF) [53].

**Table 1:** MSC trophic activities relevant to musculoskeletal therapy: mechanistic insights from in-vitro and host tissue studies

System and reference	In vitro/host	Cell sources	Observed trophic activity	Mechanistic insights
Angiogenesis [84]	IV	Human BM-MSCs; UCB-ECs	MSCs encouraged EC migration, proliferation, and tubule formation	GHK (osteonectin peptide) induces MSC-VEGF secretion
Angiogenesis [81]	IV	Human BM-MSCs (commercial); microvascular ECs	MSC culture on stiff, fibronectin-coated surfaces encouraged EC spreading/tubule formation	Actomyosin contractility increased MSC expression of proangiogenic factors (angiogenin, VEGF, and IGF)
Angiogenesis [105]	IV	Human BM-MSCs (commercial); UV-ECs	EC-MSC coculture increased MSC-myogenic and EC-PLAU, EC-FGF, and EC-NF-kB-regulated gene expression	• MSC IL-1β and IL-6 regulate EC NF-kB target genes, including P-selectin, CCL23, and CXCL2/3
				• EC TGF-β1/3 may regulate MSC myogenic differentiation
Angiogenesis [107]	IV/mouse	Human BM-MSCs (commercial); UV-ECs	• IV: EC-MSC (vs EC) cultures on degradable scaffolds expressed higher perivascular markers	IV: cocultures upregulated VEGF and ANG1 while downregulating ANG2
			• Host angiogenic and perivascular markers, except vessel diameter and density, were equivalent between EC/MSC-EC implants	
Angiogenesis [73]	IV/mouse	Human iMSCs (medium change of iPSCs); UV-ECs	• iMSC exosomes promoted EC migration, proliferation, and dose-dependent tubule formation (IV)	iMSCs induced EC expression of proangiogenic molecules, including VEGF, TGF-β1, and ANG1
			• Exosome treatment correlated with modest functional improvement, better perfusion and tissue damage scores, increased CD31/CD34^+^ cells	
Angiogenesis (hindlimb ischemia) [22]	Mouse	Mouse AD-MSCs (plastic adherence); BM-MSCs (plastic adherence); BM-iMSCs (immunodepletion)	• BM-MSCs maximally decreased inflammatory cell invasion	IV: BM-MSCs expressed the highest levels of tested chemokines, vessel stabilizing, and matrix-remodeling factors
			• MSCs were associated with smaller lesions, more mature neovascularization, and increased perfusion	
Neurovascular system (fibrin conduit, resection) [116]	Rat	Human AD-MSCs (plastic adherence); DRG; UV-EC	• Medium cocktail-stimulated MSCs enhanced DRG neurite extension and EC-tubule formation	Stimulated MSCs produced increased VEGF, ANG1, NGF, BDNF, and GDNF
			• Stimulated and unstimulated MSCs encouraged neurite extension	
Neurogenesis [167]	IV	Rat BM-MSCs (plastic adherence)	Spinal cord tissue–MSC coculture supported neurite outgrowth	Cocultured MSCs produced NGF, BDNF, and GDNF, maximally supporting neurite extension
Neurogenesis (spinal nerve ligation) [123]	Rat	Rat BM-MSCs (commercial)	MSC-treated rats displayed decreased hyperalgesia and increased pain threshold	TUBB3^–^, GFAP^–^, and αSMA^–^ and STRO1^+^ MSCs engrafted into DRGs
Neurogenesis (sciatic crush) [124]	Mouse	Human AD-MSCs and AM-MSCs (commercial)	• AM-MSC-treated groups exhibited higher recovery, coordination, and perfusion scores (4 weeks)	Nerves injected with AM-MSCs versus AD-MSCs or PBS produced more ANG1, FGF1, IGF1, and VEGFA
			• MSCs localized in the epineurium and perivascular area	
Distraction Osteogenesis (DO) [59]	Mouse	Human BM-MSCs (commercial)	• MSC and MSC-CM accelerated DO healing	• IV: IL-3/IL-6/CCL5/SDF1 recruited mononuclear cells, contributed to enhanced mineralization
			• MSC-CM recruited more vessels	
				• MCP1/MCP3 but not SDF1 were critical for SC-CM osteogenic activity
Osteogenesis [168]	Mouse	Human AD-MSCs and BM-MSCs; UCB-ECs	• MSC-EC cotransplantation increased MSC engraftment	PDGFBB/PDGFRβ receptor activity regulates MSC engraftment and differentiation in the presence of ECs
			• Cotransplantation restricted MSC multipotency, enhanced MSC source-related differentiation abilities, and maintained MSC proliferation capacity	
Osteoporosis (lupus associated) [60]	Mouse	Human BM-MSCs and DP-MSCs	• MSC injections improved osteoporosis-related bone scores	IL-17 removal following MSC injection maintains osteoclast immaturity
			• MSCs lowered osteoclast differentiation (IV)	
Osteogenesis [169]	Rat	Rat BM-MSCs (centrifugation and plastic adherence)	Fibrin-loaded MSC recruited host macrophages to fill long bone defect by 4 weeks	Implanted MSCs increased early expression of VEGF and decreased later expression of CD45, IL-6, IL-1β, TNF-α, and IL-10
Osteogenesis, chondrogenesis, angiogenesis [170]	IV	Human BM-MSCs (density gradient) and human embryonic stem cell MSCs (medium/substrate changes); human aortic ECs	MSC-EC cocultures proliferated and exhibited higher expression of mesenchymal differentiation transcription factors	EC-produced ET1 activates MSC AKT, driving osteogenic and chondrogenic capacities
Chondrogenesis [95]	IV	Human BM-MSCs (density gradient)	• MSCs and/or chondrocytes in fibrin gels exhibited superior mechanical properties to those cultured with OA cartilage explants	IL-1β and IL-6 decreased COL production versus control cultures, except in chondrogenic cultures at longer culture times (4 weeks)
			• COLI/II/III production reduced in OA cartilage–MSC or chondrocyte–MSC cocultures	
Chondrogenesis [93]	IV	Human BM-MSCs; Human OA primary chondrocytes; bovine primary chondrocytes	FGF1 caused chondrocyte proliferation	• FGF1 was concentrated in places where MSCs contacted chondrocytes
Tenogenesis (enzymatic lesion) [152]	Horse	Horse AD-MSCs	Lesions were smaller, more vascularized, and less cellular when treated with platelet concentrate-injected MSCs	• Greater amount of RNA was recovered from the MSC-treated group
				• No difference in anabolic and tendon-specific gene expression observed
Musculogenesis (dystrophin/utrophin) [135]	IV	Mouse quickly and slowly adhering MSCs (non-myogenic﻿ nmMSCs and MPCs), dKO)	• dKO-MPC-dKO-nmMSC co-culture decreased global myogenic markers	Soluble frizzled-related protein-1 and active β-catenin encouraged nonmyogenic differentiation of dKO-nmMSCs in gastrocnemius tissues
			• dKO vs. WT-nmMSCs differentiated more efficiently along osteogenic and adipogenic lines with donor age	
Musculogenesis (myofibroblast proliferation) [138]	IV	Human AD-MSCs and BM-MSCs (commercial); Dupuytren’s disease-derived myofibroblast (DDMF)	• AD-MSCs (similar to normal skin-derived fibroblasts) decreased while BM-MSCs increased DDMF co-culture contractility	AD-MSC/myofibroblast cocultures exhibited decreased COLI and αSMA
			• AD-/BM-MSCs inhibited myofibroblast proliferation	
			• AD-MSC effects were strongest with direct or indirect contact	
Musculogenesis (dystrophin) [160]	Mouse	Human (STRO1^+^) DP-MSCs; human (c-Kit^+^) amniotic fluid MSCs	• MSCs differentiated in the presence of C2C12-formed myotubes (IV)	Demethylation was critical for IV myogenic differentiation
			• MSCs differentiated most efficiently with C2C12-CM	
			• All differentiated MSCs engrafted and improved muscle histology	
Musculogenesis [137]	IV	Mouse BM-MSCs (centrifugation and plastic adherence)	MSC-CM stimulated myoblast and satellite cell proliferation and migration, activated satellite cells, inhibited myofibroblast differentiation	MSC MMP-2/9 and TIMP-1/2 support myogenic differentiation

*AD*, adipose-derived, *AM* amniotic membrane, *BM*, bone marrow, *CM* conditioned medium, *dKO* double knockout, *DP* dental pulp, *DRG* dorsal root ganglia, *EC* endothelial cell, *iMSCs* MSCs generated from induced pluripotent stem cell (*iPSC*) lines via medium change, *IV* in vitro, *MMP* matrix metalloproteinase, *MPC* multipotent cell, *MSC* mesenchymal stem cell, *SC* stem cell, *TIMP* tissue inhibitor of metalloproteinase, *UCB* umbilical cord blood, *UV* umbilical vein

In addition to some of the classic chemotactic growth factors and molecules already mentioned (HGF, PDGF, and bFGF), MSCs are strongly influenced by the binding of CXCL12 (SDF1) to CXCR4 [54]. Embryonic muscle growth and adult muscle repair are thought to be heavily influenced by MMP-10-regulated CXCL12 stimulation of MSC migration [55, 56]. Additionally, MSCs express a variety of receptors, including various integrins and selectins, that allow extravasation at repair sites [57].

**Fig. 1:**
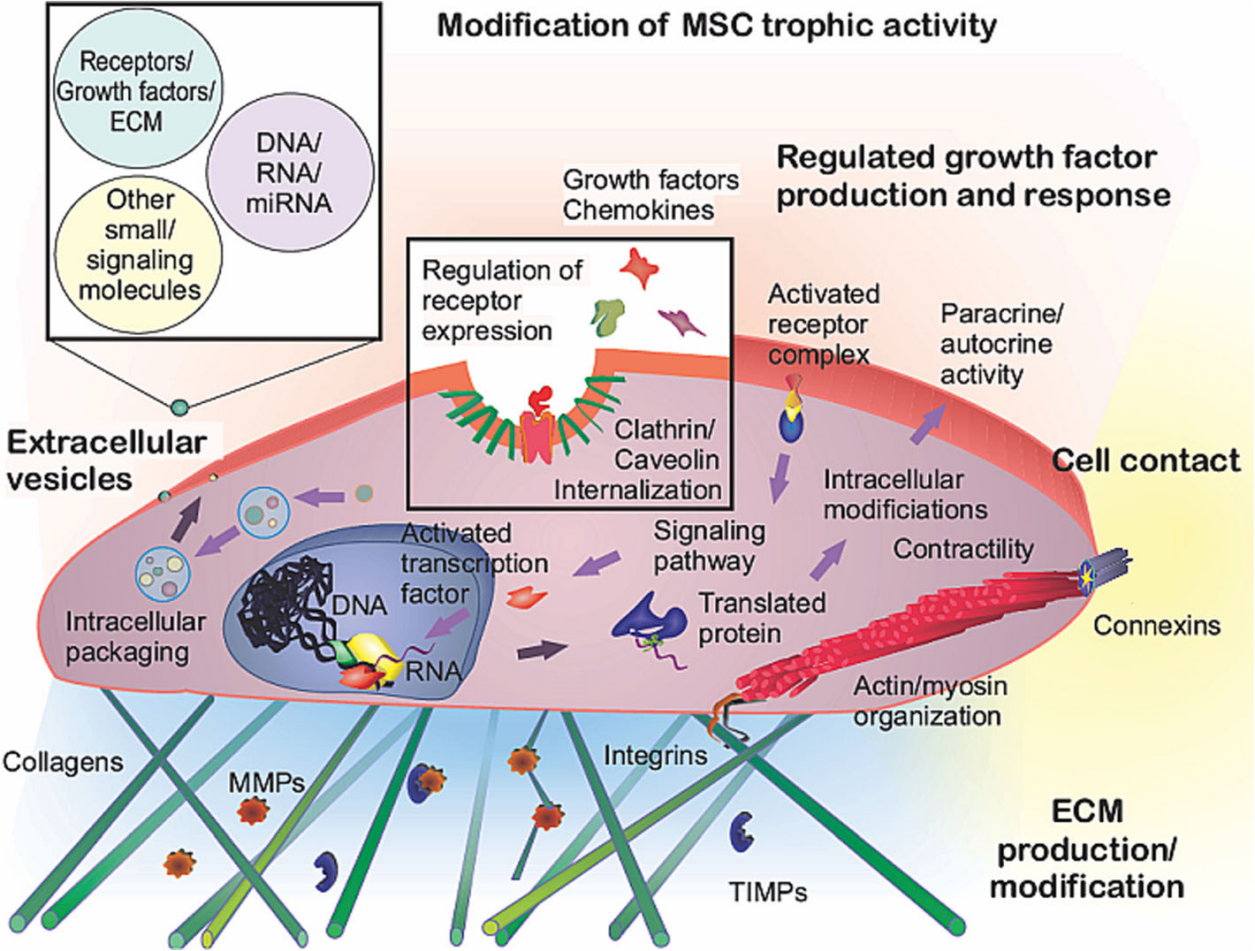
MSC trophic mechanisms depend on MSC interactions with and modification of the local environment. MSC trophic functions can be both achieved and altered through dynamic ECM–cytoskeletal interactions, cell–cell contacts, and soluble and transcription factor signaling. *ECM* extracellular matrix, *miRNA* microRNA, *MMP* matrix metalloproteinase, *MSC* mesenchymal stem cell, *TIMP* tissue inhibitor of metalloproteinase

Clues to the mechanisms of MSC trophic activities (Fig. 1) can also be found in the extensive work done in other fields, particularly exploration of the stem cell secretome in the cardiac field [58]. Identified factors include adrenomedullin, angiogenin, fibroblast growth factor-2 (FGF2), CXCL12, cistatin C, cysteine-rich angiogenic inducer 61 (Cyr61), Dickkopf-related proteins (Dkk), hepatocyte growth factor (HGF), insulin-like growth factor (IGF), IL-1, IL-6, pigmented epithelium-derived factor (PEDF), placental growth factor (PLGF), SDF1, TSG6, VEGF, MMP-2, tissue inhibitor of metalloproteinase-1 (TIMP-1), TIMP-2, secreted frizzled related protein-2 (SFRP-2), thrombospondin-1, and tenascin C [58]. Belying their osseous origin, CM of BM-MSC appears enriched in molecules typically secreted by or influencing osteoblasts, including decorin, osteoprotegerin, Dkk-3, receptor activator of nuclear factor-kB (RANK), osteopontin, and CCL5; inflammatory factors maximally produced by BM-MSCs include CCL2, TIMP-2, IL-6, IL-7, IL-3, MMP-7, chemokine (C-X-C) receptor-16 (CXCR16), and MMP-10. CCL2 and CCL7 produced by BM-MSCs appear to strongly influence nascent bone formation [59, 60]. Recent work also suggests that AD-MSC, BM-MSC, and dental pulp stem cell-secreted CXCL14 and CCL2 help to recruit CXCR4^+^ cells and chemokine (C-C) receptor-2^+^ (CCR2^+^) vessel-associated cells, without inducing proliferation [61]. Besides these influential but potentially short-lived proteins, some MSCs secrete EVs which may contain any number of influential molecules, protected from systemic degradation by virtue of their natural, membrane-bound packaging [62–65].

In many cell types, EVs of varying sizes, including ectosomes/exosomes and microvesicles/microparticles, were derived from either cytoplasmic protrusions or lipid raft internalization and subsequent endosomal fusion with the plasma membrane [66–69]. These 30 nm–1 μm vesicles may be studded with multiple proteins, usually tetraspanins, and filled with a combination of proteins, lipids, and copious amounts of mRNA and microRNA (miRNA), particularly miR22 and miR-19a [58]. EC and cancer cell-derived microparticles may potently increase MSC NF-kB activity, stimulating local trophic support [70]. Gap junctions, formed via connexins, are another avenue allowing direct cell–cell communication, with strong evidence for membrane and (to a lesser extent) cytoplasmic exchange between MSCs and ECs [71].

Likely mediated through EC-stimulated VEGF production, CXCR4-enriched exosomes derived from MSCs generated from induced pluripotent stem cell (iPSC) lines via medium change (iMSCs) improved recovery from myocardial infarction [72]. In work addressing hindlimb ischemia in mice, iMSC exosomes were associated with a higher number of CD31^+^ and CD34^+^ cells in damaged muscle tissue as well as increased EC secretion of VEGF, TGF-β1, and angiogenin, suggesting enhanced vascular recruitment by vesicles alone [73]. Another group generated iMSCs through TGF-β-pathway inhibition and medium changes; those iMSCs, surprisingly, did less to promote cancer than BM-MSCs, appearing to express and produce lower amounts of several of the inflammatory and differentiation factors (particularly TGF-β receptor-2 and, interestingly, hyaluronan (HA)) when cultured with various types of cancer cells [74].

In a recent study, Baglio et al. [75] characterized the RNA contents of exosomes obtained through ultracentrifugation, and found that exosomes were enriched for tRNA, in particular tRNA CTC. Their work further suggested that the differentiation state of a MSC might be deduced by the content of its exosomes, particularly the presence of full-length tRNA and tRNA long fragments, consistent with a stem-like state of the cells [76]. miRNA loading within vesicles is not random, as dexamethasone treatment of both C2C12 cells and diabetic rats increased the concentration of miR-23a and miR-182 in collected microvesicles and urine, respectively [77], thus providing MSCs with a dynamic way to influence and respond to their microenvironment. Microvesicles may also suppress the infiltration of macrophages into damaged tissues [75]. However, the relevance of vesicles/microvesicles to classical and clinically approved MSCs is questionable, because a different group noted that MSCs produced much fewer vesicles than immortalized ESC-derived MSCs [78]. The group offered an immortalization strategy that would maximize the yield of MSC-produced vesicles, should they prove effective in the clinic.

## Altering MSC activity prior to bone or cartilage implantation

Because “plain” MSC implantation has faced such varied success in the clinic, the next generation of MSC-based strategies seeks to harness and direct MSC trophic activities. It was observed that substrate composition and stiffness, sensed through various integrins, could influence the expression of myogenic factors [79]. Substrate stiffness, known to affect the differentiation of MSCs, acting possibly through regulation of alpha-smooth muscle actin (αSMA), could also play a role in the eventual differentiation capacity of culture-expanded MSCs [80]. Substrate stiffness, in turn, affects MSC trophic properties; stiff (40 kPA) polyacrylamide gels coated with fibronectin induced proangiogenic factor secretion by BM-MSCs [81]. Through internalization and recycling of focal adhesions and receptors, caveolins play an intriguing role in MSC sensing of both substrate stiffness and surrounding soluble signals, particularly in vascular, muscular, and osteogenic settings [82]. Alternatively, hydrogels made from autologous plasma may also help to temporarily concentrate either AD-MSCs or AD-MSC-CM at the injury site [83].

The ability of MSCs to self-generate abundant collagenous extracellular matrix (ECM) may partially explain some of the positive effects witnessed in some joint degeneration trials involving MSCs [27]. Medium supplements or additional modifications to the substrate to mimic other ECM molecules or bioactive factors, such as osteonectin, may further increase MSC secretion of bioactive molecules (FGF2, CCL5, and VEGF), supporting native cell migration and differentiation [84].

The milieu present in culture serum appears to strongly influence the fate of cultured cells, more so than any single exogenous supplement [85]. One method to both encourage MSC trophic activity as well as ease immunological concerns in the clinic could be to utilize autologously derived cell culture supplements such as platelet lysate for autologous MSC expansion and culture [86]. Platelet-rich plasma, for example, may protect cartilage from injury by enhancing collagen II (COLII)/aggrecan (AGN) expression and suppressing MMP-3, COX2, iNOS, and associated NO and PGE2 production [87]. Once established in vitro, however, the role of serum becomes less clear; while serum content affects MSC gene expression and growth rate, inherent multipotency and stem cell marker surface expression do not appear to be affected by the absence of serum [88]. For that reason, MSC-CM concentration and injection may sidestep issues of autophagy [89] or apoptosis of MSCs upon in-vitro expansion [13].

Culture under hypoxia is another attractive method to increase initial MSC production of trophic factors. Under hypoxia, hypoxia inducible factor-1 alpha (HIF1α) expression increases, driving VEGF and other proangiogenic, antiapoptotic, and antioxidant molecules [90]. Under certain circumstances, hypoxic MSCs may serve to prevent harmful fibrosis through HGF production, TGF-β1/COLII, and IL-1β downregulation, and fibronectin expression [91]. Work in vitro with chondrocytes has specifically identified HGF as an antifibrotic agent released by AD-MSCs [92]. Furthermore, FGF1 secreted by MSCs in contact with chondrocytes may stimulate the proliferation of and help to preserve the function of chondrocytes [93, 94].

In-vitro evidence suggests that the addition of BM-MSCs to OA cartilage may initially increase IL-1β and IL-8 production but ultimately reduce the amount of soluble glycosaminoglycan (GAG) released by the cartilage over time, making the exact influence of MSCs on cartilage structure unclear [95].

Reported pain relief associated with the introduction of MSCs may be the result of immunomodulation but was by no means universal [36]. In-vitro studies suggest that exposure of MSCs to chondrocytes may induce expression of MSC major histocompatibility complex (MHC) I/II and other costimulatory molecules [96].

Pretreatment of MSCs, especially with IFN-γ to prime their immunosuppressive activity, may result in decreased MSC-associated tissue degradation [97]. Possibly in response to IL-1α, MSCs exposed to platelet lysate have the capacity to encourage a proinflammatory/M1 or proresolving/M2 macrophage phenotype through GM-CSF and PGE2 activity, respectively [53, 98]. Growing evidence suggests that the native, inflamed cartilage environment may both trigger the release and limit the effectiveness of MSC-anti-inflammatory PGE2 [99]. MSCs may recruit CD4^+^ T cells, which can also play a role in increasing local osteogenic activity [100]. Care must be taken with extensive culture, because MSCs may gain genetic abnormalities and lose some ability to differentiate, promoting senescence [88].

## Vascular/inflammation regulation

Several key targets of regenerative medicine therapies, including restored nerve and muscle function and the previously discussed bone repair, rely heavily on the influence of vascular cells [101]. Consequently, MSC effects on blood vessel cells have long fascinated researchers. Recent work has begun to elucidate mechanisms by which MSCs may affect blood vessel morphology and function. Chen et al. [102] reported that MSC-produced HGF, upon interaction with ECs in coculture and, to a lesser extent, via paracrine signaling, caused an increase in EC cadherin and F-actin remodeling, thereby decreasing EC permeability [90, 103, 104]. This and earlier work with ECs suggests that MSC–EC interactions may temporarily restrict both the physical clearance of MSCs and the invasion of inflammatory cells. Once in the bloodstream, MSC inhibition of NF-kB, perhaps through IL-10 or other factors, may decrease the binding of monocytes to the endothelium, further decreasing inflammation at wound/MSC injection sites [45]. This temporary pause in the battle with chronic inflammation may explain some of the positive results seen with MSCs.

Following a more classical approach focused on EC motility and activity, VEGF, ANG, and NF-kB pathways have all been implicated in regulating angiogenesis. Recent work suggests that, in the short term, the NF-kB pathway may control EC response through BM-MSC-produced IL-6 and IL-1β; following this NF-kB activation, ECs activate P-selectin, producing CCL23, CXCL2, and CXCL3. In turn, MSCs showed signs of early differentiation towards a smooth muscle phenotype in coculture, influenced by TGF-β1 and TGF-β3 [105]. BM-MSC production of VEGF may be stimulated by IL-8, either through paracrine or autocrine mechanisms [106]. In turn, BM-MSCs may stabilize ECs by upregulating ANG1, thereby downregulating EC proliferation [107]. Conversely, the interaction of MSCs with ECs, particularly through endothelin 1 (ET1) and PDGFB, may prime cells to survive transplantation and differentiate more easily upon reimplantation [108].

## Neural support

MSCs have long been known to support nerve growth through the support of Schwann cells, secretion of neurovascular factors (including FGF2 and VEGF-A), and, possibly, transdifferentiation into Schwann-like cells. Combined with varying types of biocompatible and bioactive materials, such as poly-lactic acid (PLA), polycaprolactone (PCL), polyurethane (PU), polyethylene (PE), and silicone (for strength) and COLI, HA, and so forth (for bioactivity), several groups have observed enhanced nerve extension and functional improvements in a range of animal models [109, 110]. The most recent work refines previous findings that guidance fibers of particular spacing and architecture may aid MSCs in further accelerating the nerve healing process [111–113]. AD-MSCs, at sufficient density, secrete brain-derived neurotrophic factor (BDNF) in response to autocrine IFN-β [114]. Stimulating cocktails that increase cyclic-adenosine monophosphate (cAMP) and include retinoic acid (pretreatment), FGF2, PDGFAA, and different forms of neuregulin have been shown to increase neurite outgrowth in vitro and nerve extension after injury in vivo [115]. In addition to neurotrophic BDNF, nerve growth factor (NGF), and glial cell line-derived neurotrophic factor (GDNF), as well as angiogenic VEGF and ANG1 identified in many other experiments, recent work identified the antiapoptotic activity of AD-MSCs, possibly by decreasing neuronal c-jun [116]. As mentioned in previous sections, such pretreatment is relatively common in nonclinical work, and may become de rigeur as new progenitor cell sources are explored for musculoskeletal therapies [117]. Crucially, it appears that MSCs should not be directly injected intrathecally for early spinal cord repair, as the subsequent inflammation seemed to prevent MSC migration to neuronal injury sites [118]; later injection may prove beneficial [119].

Ciliary neurotrophic factor (CNTF) is a particularly well known neuroprotective factor produced by MSCs. While the factor has potent therapeutic effects on nerve apoptosis, neuroinflammation, and neuronal proliferation, it has been linked with altered metabolism (due to neurogenesis in the hypothalamus as well as direct action on adipocytes) when administered systemically and may negatively affect osteoblast differentiation and mineralization [120–122]. GDNF, another potent neurotrophic molecule often produced by MSCs, may help to ease allodynia and hyperalgesia experienced in dorsal root ganglia sensory nerves [123]. Amniotic membrane-derived MSCs expressed more ANG1, FGF1, IGF1, and VEGFA (but not FGF2) than AD-MSCs in a mouse sciatic nerve injury trial [124].

While CM from cells treated under hypoxic and normoxic conditions both increased the observed number of differentiating neurons in vitro, hypoxia-cultured Wharton’s Jelly (WJ)-derived MSCs upregulated thymosin B and eukaryotic elongation factor (EF2) and may have contributed to a slight increase in total neuron maturity [125]. WJ-MSCs under normoxic conditions were shown to produce PDGFAA, HGF, TGF-β2, IL-6, IL-8, IL-1ra, CCL5, CCL2, and CXCL10 at much larger concentrations than BM-MSCs and AD-MSCs [126]. Whatever the mechanism for neural support, tissue response to neurological directives is critical to the ultimate utility of the repaired nerve.

## Muscles and miscellany

Intriguing results suggest that MSCs derived from less traditional sources could one day be utilized therapeutically. One readily available source for MSCs could be skeletal muscles. Our research group has worked extensively with blast-traumatized muscle-derived multipotent cells [127–129]. This particular type of muscle-derived multipotent cells is especially attractive therapeutically due to its relative abundance and ease of isolation [130] as well as neurotrophic activity [131]. MDSCs should be used cautiously when attempting to rebuild musculoskeletal tissues, because several groups have identified populations that seem predisposed to mineralize ectopically [132–134], especially in the presence of muscular genetic abnormalities [135]. Growth factor coinjection might attenuate this ectopic bone formation, as growth hormone–insulin-like growth factor-1 (GH-IGF1) activity promotes muscle cell proliferation, regulates muscle fiber size and type, controls osteoblast proliferation and differentiation, inhibits osteoclast activity, stimulates renal conversion of 25-OH-vitamin D_3_, and controls phosphate reabsorption [136]. By contrast, this matrix-modifying MSC activity may help to attenuate disease severity and ultimately contribute to useful muscle mass [137]. Harmful proliferation and contraction of myofibroblasts, as occurs in Dupuytren's contracture, may be attenuated in the presence of the CM of both AD-MSCs or BM-MSCs as well as the physical presence of AD-MSCs (but not BM-MSCs) [138]. BM-MSCs appear to contribute to pathological myofibroblast proliferation while AD-MSCs appear to inhibit the activity slightly [138]. MMP-2 and MMP-9 are required for efficient skeletal muscle regeneration and are enhanced by mouse BM-MSCs/MSC-CM along with reduced TIMP-1/2 levels. Muscle cell motility may also be encouraged by BM-MSC-secreted MMP-2 [139].

Aside from their ECM-modifying properties, the immunomodulatory properties of MSCs are intriguing from a therapeutic standpoint but must be used carefully, because MSC treatment, concurrent with a *Staphylococcus aureus* infection, was shown to increase the severity of bone loss, despite increased MSC proinflammatory cytokine expression, in an osteomyelitis model [140]. Conversely, encouraging results were recently published from a small idiopathic osteonecrosis trial in Japan, where BM-MSCs were isolated, cultured for 2 weeks, and returned to osteonecrotic patients along with tricalcium phosphate chips (Osferion) and tricortical iliac crest bone [141]; after a 12-week rehabilitation program, all patients reported reduced pain and increased physical function with no serious adverse events reported in the study [142]. The likelihood of MSC engraftment being the cause for the recovery is low, however, as MSCs have been found to migrate towards apoptotic cells, via HGF signaling, but not HGF produced in the presence of necrotic cells [143].

Evidence of MSC trophic efficacy has generated intense excitement in clinically focused research. This excitement is evident in the increasing number of reviews examining MSC trophic properties. Marked therapeutic successes will likely hinge on technological and computational advancements that allow dynamic, high-resolution, and quantitative observation of MSC–ECM, MSC–paracrine, and MSC–cellular interactions to better define the appropriate perspective on the true activity of MSCs.

## Conclusions

The application of allogeneic and autologous MSC therapies for the treatment of diseases and dysfunctions of multiple musculoskeletal tissues has received increasing attention. Exciting in-vitro and in-vivo investigations on tendon [117, 144, 145], meniscus [146–148], and ligaments [149, 150] have been reported, along with the use of autologous products such as platelet-rich plasma/plasma lysate [151]. Studies using larger, clinically relevant animal models are both underway and necessary before human clinical trials can be developed [152].

This review has primarily explored secreted trophic factors produced by MSCs. A whole host of therapies are dedicated to engineering or modifying the physical environment and ECM of MSCs to affect their therapeutic potential. A recently developed approach attempts to anchor cells to the collagenous tissue matrix by engineering collagen anchors [153], to promote local action of MSCs and minimize their systemic loss to the lungs, liver, and spleen. Changes in substrate composition (especially the presence of collagen) and stiffness may expand the potential applications of MSC therapies to include muscle volume loss through stimulation of muscle-resident progenitor cells [134, 154, 155]. Local ECM modifications are known to affect MSC differentiation potential [156, 157] and are beyond the scope of this review. Through continuing advancements in genetic engineering, MSCs may eventually be used to treat genetic musculoskeletal conditions, including osteogenesis imperfect [158] and Duchenne’s muscular dystrophy [159, 160]. Careful selection of the therapeutic cells, taking into account subtle tissue source-related differences, may be the key to successful clinical dystrophy therapies [35]. To prove their efficacy in the clinic, these potential treatments will need to be tested in well-controlled studies to assess physical functions for an extended period of time [27].

New or more precise modes of MSC trophic activity may be discovered by adopting contemporary analytical technologies to evaluate and compare genomic, transcriptomic, proteomic, metabolomic, and secretomic profiles, exemplified by the great strides that have been made in genetic and metabolic diseases [161–164]. Lessons learned from previous iterations of MSC therapies and clinical drug trials should overcome some of the regulatory and therapeutic hurdles to MSC use [2, 32, 33]. It is also noteworthy that while genetic engineering of iPSCs may hold the answer to unlimited numbers of perfectly-tuned stem cells, managing the safety concerns of iPSCs and negotiating the patent landscape of this saturated market will be highly challenging [165]. Another major challenge is the uncertainty in terms of biological responsiveness of the diseased tissue, because evidence in several fields suggests that ischemic tissue may be incapable of responding to MSCs [166]. Finally, each of these avenues should be explored while juggling the needs for rigorous science, proven therapeutic efficacy, regulatory approval (e.g., by the Food and Drug Agency) (and thus reproducibility) of a final therapy, and cost/benefit for the patient.

## Abbreviations

AD-MSC: Adipose-derived mesenchymal stem cell
AGN: Aggrecan
Ang: Angiopoietin
BDNF: Brain-derived neurotrophic factor
BM: Bone marrow
BMI: Body mass index
BMP: Bone morphogenetic protein
cAMP: Cyclic adenosine monophosphate
CCR/L: Chemokine (C-C) receptor/ligand
CD: Cluster of differentiation
CM: Conditioned medium
CNTF: Ciliary neurotrophic factor
COL: Collagen
COX: Cyclooxygenase
CPC: Chondrocyte progenitor cell
CSPC: Cartilage-derived stem/progenitor cell
CXCR/L: Chemokine (C-X-C) receptor/ligand
Cyr61: Cysteine-rich angiogenic inducer 61
Dkk: Dickkopf-related proteins
EC: Endothelial cell
ECM: Extracellular matrix
EF2: Eukaryotic elongation factor
ET: Endothelin
EV: Extracellular vesicle
FGF: Fibroblast growth factor
Foxp3: Forkhead box p3
GAG: Glycosaminoglycan
GARP: Glycoprotein A repetitions predomain
GDNF: Glial cell line-derived neurotrophic factor
GH-IGF: Growth hormone-insulin-like growth factor
GM-CSF: Granulocyte macrophage colony-stimulating factor
HA: Hyaluronan
HGF: Hepatocyte growth factor
HIF: Hypoxia inducible factor
HLA: Human leukocyte antigen
IDO: Indoleamine 2,3-dioxygenase
IFN: Interferon
IGF: Insulin-like growth factor
IL: Interleukin
iMSC: MSC generated from induced pluripotent stem cell (iPSC) lines via medium change
iNOS: Nitric oxide synthase
LIF: Leukemia inhibitory factor
LRRC32: Leucine-rich repeat containing-32
MDSC: Muscle-derived stem cell
MHC: Major histocompatibility complex
miRNA: MicroRNA
MMP: Matrix metalloproteinase
mRNA: Messenger RNA
MSC: Mesenchymal stem cell
NF-κB: Nuclear factor-kappa B
NGF: Nerve growth factor
NO: Nitric oxide
OA: Osteoarthritis
PCL: Polycaprolactone
PDGF: Platelet-derived growth factor
PE: Polyethylene
PEDF: Pigment epithelium-derived factor
PG: Prostaglandin
PLA: Poly-lactic acid
PLGF: Placental growth factor
PU: Polyurethane
RA: Rheumatoid arthritis
RANK: Receptor activator of nuclear factor-κB
Rgs: Regulator of G-protein signaling
SDF: Stromal-derived factor
SFRP: Secreted frizzled related protein
TGF: Transforming growth factor
TIMP: Tissue inhibitor of metalloproteinase
TNF: Tumor necrosis factor
TSG: Tumor necrosis factor-inducible gene
UCB: Umbilical cord blood
UV: Umbilical vein
VEGF: Vascular endothelial growth factor
WJ: Wharton’s jelly
αSMA: Alpha smooth muscle actin
